# Targeting microbial pathogens by expression of new recombinant dermaseptin peptides in tobacco

**DOI:** 10.1002/mbo3.837

**Published:** 2019-03-25

**Authors:** Mitra Khademi, Farhad Nazarian‐Firouzabadi, Ahmad Ismaili, Reza Shirzadian Khorramabad

**Affiliations:** ^1^ Agronomy and Plant Breeding Department, Faculty of Agriculture Lorestan University Khorramabad Iran; ^2^ Agricultural Biotechnology Department, Faculty of Agricultural Sciences University of Guilan Rasht Iran

**Keywords:** *Agrobacterium rhizogenes*, antimicrobial peptide, chitin‐binding domain, expression, hairy roots

## Abstract

Dermaseptin B1 (DrsB1), an antimicrobial cationic 31 amino acid peptide, is produced by *Phyllomedusa bicolor*. In an attempt to enhance the antimicrobial efficacy of DrsB1, the DrsB1 encoding 93 bp sequence was either fused to the N or C terminus of sequence encoding chitin‐binding domain (CBD) of *Avr*4 gene from *Cladosporium fulvum*. Tobacco leaf disk explants were inoculated with *Agrobacterium rhizogenes* harboring pGSA/CBD‐DrsB1 and pGSA/DrsB1‐CBD expression vectors to produce hairy roots (HRs). Polymerase chain reaction (PCR) was employed to screen putative transgenic tobacco lines. Semi‐quantitative RT‐PCR and western blotting analysis indicated that the expression of recombinant genes were significantly higher, and recombinant proteins were produced in transgenic HRs. The recombinant proteins were extracted from the tobacco HRs and used against *Pectobacterium carotovorum*, *Agrobacterium tumefaciens*, *Ralstonia solanacearum*, and *Xanthomonas campestris* pathogenic bacteria and *Alternaria alternata* and *Pythium *sp. fungi. Two recombinant proteins had a statistically significant (*p* < 0.01) inhibitory effect on the growth and development of plant pathogens. The CBD‐DrsB1 recombinant protein demonstrated a higher antibacterial effect, whereas the DrsB1‐CBD recombinant protein demonstrated greater antifungal activity. Scanning electron microscopy images revealed that the structure of the fungal mycelia appeared segmented, adhered to each other, and crushed following the antimicrobial activity of the recombinant proteins. Due to the high antimicrobial activity of the recombinant proteins against plant pathogens, this strategy can be used to generate stable transgenic crop plants resistant to devastating plant pathogens.

## Graphical abstract

Harnessing a chitin‐binding domain from *Cladosporium fulvum* *Avr*4 effector protein as fusion to the Dermaseptin B1 peptide enhances the antimicrobial activity of the peptide toward microbial pathogens.

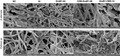

## 1 INTRODUCTION

Plant pests and diseases are among the main factors reducing the production of agricultural products and diminishing their quality and yield as well as threatening food safety (Oerke, 2006). In developing countries, it is estimated that pests and diseases decrease the yield of crop plants by 30%–40% (Flood, 2010). Chemical control is often used to combat devastating plant pathogens. However, given the negative effects of chemical control on human health and the environment, and emergence of resistance by pathogens, it is necessary to employ safer and more sophisticated methods to cope with plant pathogens (Vidaver, 2002).

Plants activate their immune‐defense system when pathogens attack (Nguyen, Haney, & Vogel, 2011; Zasloff, 2002, 2006). For instance, following infection, plants express chitinases, glucanases and produce defensins and other antimicrobial peptides (AMPs) to fight pathogens (Bruce & Pickett, 2007). A broad spectrum of living organisms produces AMPs (Nguyen et al., 2011; Zasloff, 2002, 2006). AMPs, as an important part of host immune system, play a pivotal role in plant resistance toward pests and pathogenic microbes (Nguyen et al., 2011; Zasloff, 2002, 2006). Despite the considerable diversity among plant defensins, plant pathogens can still inflict considerable damage to plant yield and quality (Portieles et al., 2010).

Among more than 3,053 peptides listed in the antimicrobial peptide database so far (23‐01‐2019), peptides belonging to the Dermaseptin family show high antimicrobial activity and are mostly lytic to microbial pathogen (Reddy, Yedery, & Aranha, 2004). Dermaseptins are 23–35 amino acid long peptides produced and secreted by the skin glands of a number of frog families (Tossi, Sandri, & Giangaspero, 2000). Dermaseptins show a broad‐spectrum inhibitory effect against both gram‐negative and gram‐positive bacteria (Navon‐Venezia, Feder, Gaidukov, Carmeli, & Mor, 2002; Osusky, Osuska, Kay, & Misra, 2005; Yaron, Rydlo, Shachar, & Mor, 2003), yeasts (Coote, Holyoak, Bracey, Ferdinando, & Pearce, 1998), protozoa (Hernandez et al., 1992), and fungi (De Lucca, Bland, Jacks, Grimm, & Walsh, 1998).

Fungal cell wall plays a crucial role in fungal pathogenesis since it acts as the major interface with the host immune system (Hopke, Brown, Hall, & Wheeler, 2018), and fungal mutants affected in the biosynthesis of cell wall oligosaccharides are severely affected in their growth and pathogenicity (Bowman & Free, 2006). Chitin is one of the main structural oligosaccharides of the cell wall in various pathogenic fungi, playing a role of an important elicitor of innate defense responses in plants (Sánchez‐Vallet, Mesters, & Thomma, 2015). Through their hydrolytic activities, plant chitinases hydrolyze cell wall chitin eventually leading to cell death (Latgé, 2010; Latgé & Beauvais, 2014; Thomma, Nürnberger, & Joosten, 2011). To fight back, fungal pathogens employ various strategies, including changes in cell wall oligosaccharides, secreting effectors, and masking cell wall components (Fujikawa et al., 2012; Latgé & Beauvais, 2014). For example, *Cladosporium fulvum* produces and secretes *Avr*4 effectors at the infection site covering the cell wall chitin inhibiting plant hydrolytic enzymes (van den Burg, Harrison, Joosten, Vervoort, & de Wit, 2006). The *Avr*4 effector has a CBD binding to the chitin in fungal cell walls, decreasing host chitinases access to fungal cell walls and thus preventing fungal cell wall degradation (van den Burg et al., 2006).

Since correct packaging, disulfide bonds formation, accumulation of multi‐subunit proteins, and posttranslational modifications are performed by plant cells accurately, plant systems are used to produce eukaryotic recombinant proteins (Daniell, Streatfield, & Wycoff, 2001; Giddings, Allison, Brooks, & Carter, 2000). However, low production levels, high extraction costs, and limitations in the degree of recombinant protein purification are among major challenges facing recombinant protein production in plants (Borisjuk et al., 1999). The use of *Agrobacterium rhizogenes*‐mediated transformation to produce hairy roots (HRs) is one of the efficient strategies to produce recombinant proteins and secondary metabolites (Borisjuk et al., 1999).

The use of genetic engineering strategies to introduce genes encoding natural and synthetic antimicrobial peptides is a new approach in engineering resistance to a broad spectrum of pathogens (Zasloff, 2002). Various recombinant proteins and peptides have so far been introduced in HRs of different plant species, and their antimicrobial properties have been tested in vitro (Aleinein, Schäfer, & Wink, 2015; Moghadam, Niazi, Afsharifar, & Taghavi, 2016; Pham, Schäfer, & Wink, 2012). For instance, an anti‐HIV protein and an anti‐tumor protein MAP30, including ribosome‐inhibiting proteins were produced in tobacco HRs (Moghadam et al., 2016). Analysis of total proteins extracted from transgenic HRs indicated a strong antimicrobial activity against both gram‐positive and gram‐negative bacteria as well as pathogenic fungi (Moghadam et al., 2016). Chahardoli, Fazeli, and Ghabooli (2018) expressed a lactoferricin encoding protein in tobacco HR culture with potent antibacterial activity against *Escherichia coli* (Chahardoli et al., 2018). Introduction of ranalexin peptide in tobacco HRs resulted in efficient production of ranalaxin in HRs with strong inhibitory effect on multidrug resistant gram‐positive and gram‐negative pathogens such as *Staphylococcus aureus*, *Streptococcus pyogenes*, and *E. coli* and on *Enterococcus *strains resistant to vancomycin, suggesting that tobacco HR culture was a suitable system to produce ranalexin and other recombinant peptides (Aleinein et al., 2015).

In this study, we showed that fusion of dermaseptin B1 (DrsB1) antimicrobial peptide to the chitin‐binding domain (CBD) of Avr4 protein from *C. fulvum* enhanced the antibacterial activity of DrsB1 peptide, suggesting that CBD might facilitate DrsB1 peptide access to the fungal plasma membrane, leading to cell membrane rupture and deformation.

## 2 MATERIALS AND METHODS

### 2.1 Expression cassettes

**Figure 1:**
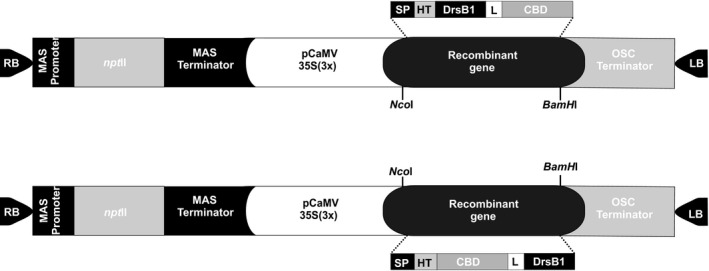
The schematic illustration of the expression vectors used for the recombinant protein production in tobacco hairy roots. MAS: Mannopine synthase, *npt* II: Neomycin phosphotransferase II, CaMV35S: Cauliflower mosaic virus 35S promoter, OSC: Octopine synthase, SP: *Avr*4 signal peptide, HT: Histidine tag (RGS‐(His)_6_), L: (EAAAK)_4_ Linker, CBD: chitin binding domain, RB: right border and LB: left border

Ninety‐three nucleotide‐long DNA sequence encoding the DrsB1 antimicrobial peptide (UniProtKB accession number P80282) was codon optimized and fused either to the C or the N terminus of the 192 nucleotides‐long sequence encoding the CBD of *Avr*4 effector gene of *C. fulvum* (GenBank accession number CAA69643.1). Two recombinant constructs were chemically synthesized and cloned in two pUC cloning vectors (Biomatik, Canada). Sequence encoding 18 amino acids long signal peptide (SP) of *Avr*4 gene was also fused to the 5′ end of the constructs to ensure secretion of recombinant proteins in the apoplastic space. Moreover, the cleavage sites of *Nco*I and *Bam*HI restriction enzymes were engineered at the 5′ and the 3′ end of the recombinant genes for cloning purposes (Figure 1). The rice chitinase helix‐forming linker (EAAAK)_4_ sequence was used to fuse DrsB1 to CBD. The pUC vectors were digested with *Nco*I and *Bam*HI restriction enzymes, and recombinant fragments were subcloned in the pGSA1285 expression vector, resulting in pGSA1285/CBD‐DrsB1 and pGSA1285/DrsB1‐CBD vectors (Figure 1). The recombinant genes were driven by cauliflower mosaic virus 35S (3×) promoter. The Arg‐Gly‐Ser‐(His)_6_ sequence was engineered after SP to identify the recombinant proteins (Figure 1).

### 2.2 *Agrobacterium* *rhizogenes*‐mediated transformation

Tobacco (*Nicotiana tabacum*) Xanthi cultivar seeds were disinfected in a detergent solution (5% sodium hypochlorite and Triton X‐100) for 10 min and then washed three times with distilled water to remove detergent residues. The seeds were then germinated on MS culture medium under 16 hr light/8 hr dark photoperiod at 24 ± 2°C. Two *A. rhizogenes *(ATCC15834) clones harboring the pGSA1285/CBD‐DrsB1 and pGSA1285/DrsB1‐CBD expression vectors were used to inoculate tobacco leaf disks. Briefly, sterilized 3‐week‐old tobacco leaf disks (1 cm^2^) were cut and put in an *A. rhizogenes *two‐day inoculation suspension for 10 min. The inoculated leaves were dried using a sterile filter paper and then placed on the hormone and antibiotic‐free MS culture medium. The inoculated leaf disks were incubated at 24 ± 2°C for 2–3 days in dark and were eventually transferred to the selective medium containing kanamycin (50 mg/L) and cefotaxime (200 mg/L) for HR induction. The explants were regularly subcultured once every 2 weeks until root formation (Tempe & Casse‐Delbart, 2012).

### 2.3 Genomic DNA extraction and screening of putative transgenic HRs

Genomic DNA was extracted from putative transgenic and control HRs, using the CTAB method (Gawel & Jarret, 1991). In order to demonstrate that the HRs are free of *A. rhizogenes* cells, the polymerase chain reaction (PCR) was performed using the *Vir*G specific primers (VirGF:5′‐CCGCGGTCAGCCGCAATTCT‐3′; VirGR:5′‐CTGCACGTCCGCGTCAAAGAAATA‐3′). The presence of the T‐DNA in HRs was confirmed by PCR amplification of the *rol*C gene by using *rol*C specific primers (rolCF:5′‐CTCCTGACATCAAAACTCGTC‐3′, rolCR:5′‐TGCTTCGAGTTATGGGTACA‐3′). Finally, to screen the putative transgenic HRs lines, the DrsB1 specific primers (DrsF:5′‐GCTAAGGCTATGTGGAAGGATG‐3′, DrsR:5′‐ATTGAGAAATAGTATCAGCAACAGC‐3′) were used. In each PCR, the initial denaturation was carried out once at 94°C for 5 min followed by 35 cycles of denaturation (at 94°C for 1 min), annealing (at 59°C, 53°C and 59°C, respectively for 30 s), extension (at 72°C for 30 s to one min), and a final extension at 72°C for 5 min.

### 2.4 Expression analysis of the recombinant genes

Total RNA was extracted following lithium chloride method (Li, Wang, Sun, & Li, 2011) followed by DNase treatment to remove genomic DNA contamination. cDNA was synthesized using a cDNA synthesis kit (Thermo Fisher Scientific Inc., #k1622). Semi‐quantitative RT‐PCR analysis was performed according to Nazarian Firouzabadi et al. (2007) employing the DrsB1 specific primers (RtDrsF: GCTAAGGCTATGTGGAAGGATG; RtDrsR: ATTGAGAAATAGTATCAGCAACAGC). The elongation factor 1α (*elf*‐1α) gene was used as an internal control (Nazarian Firouzabadi et al., 2007).

### 2.5 Total protein extraction from HRs

One‐gram HR tissue from transgenic and control lines were ground in liquid nitrogen and homogenized in a potassium phosphate buffer (50 mM at pH7, 1 mM phenylmethylsulfonyl fluoride). The homogenized samples were vigorously vortexed for 2–5 min and centrifuged at 13,000 rpm for 30 min at 4°C, and the supernatants were filtered using 0.45 µm membranes (Stone & Gifford, 1997). Furthermore, total protein was also extracted from a DrsB1‐expressing (DrsB1‐04) transgenic HR with no CBD fused as a control (Alibakhshi, 2017). The concentration of extracted proteins was measured using the Bradford method (Bradford, 1976), and proteins were stored at −20°C.

### 2.6 Purification of the recombinant proteins and Western blot analysis

To purify the expressed recombinant proteins, total protein isolated from transgenic and controls was transferred to a chromatography column containing the PrepEase Ni‐IDA resin. The column was prewashed with 300 mM NaCl and then with 50 mM NaH_2_O_4_. The purified recombinant proteins were removed from the column by rinsing the column, using 250 mM imidazole. Purified proteins were then loaded on 14% acrylamide gel and electrophoresed at 150 V. The recombinant proteins were electroblotted by using a Mini‐Protean II Multiscreen Apparatus (Bio‐Rad). The nitrocellulose blot was blocked for 1 hr, using tris buffered saline (TBS) containing 5% powdered milk. The blots were washed three times with TBS buffer and then exposed to 1:2000 dilution of mouse monoclonal anti‐His antibody at 37°C for 1 hr followed by 3,3′‐diaminobenzidine tetrahydrochloride (DAB) detection according to the manufacturer's instructions (Bollag, Edelstein, & Rozycki, 1996).

### 2.7 Antimicrobial activity of the recombinant proteins

Antimicrobial activities of the recombinant proteins were determined by studying the activity of the total protein extracted from transgenic HRs against the gram‐negative plant bacteria, including *Agrobacterium tumefaciens *(PTCC 1654), *Pectobacterium carotovorum *subsp*. carotovorum* (PTCC 1675), *Ralstonia solanacearum *(ATCC 11696), and* Xanthomonas campestris *(PTCC 1473) using the disk diffusion method (Bauer, Kirby, Sherris, & Turck, 1966; Mangena & Muyima, 1999). Bacterial cultures were provided by the Department of Plant Protection, Faculty of Agriculture, Lorestan University, Iran. Briefly, 100 µl of 21 hr old culture of the bacterial suspensions of half‐MacFarland (1.5 × 10^8^ cfu/ml) was poured onto the surface of the nutrient agar culture medium and spread by using a sterilized cotton swab. Next, 50 µl of the filter sterilized extracted fusion proteins (60 μg/ml) were added to each of the sterile 6‐mm disks. The disks containing the recombinant proteins were then placed on the bacterial culture medium. The Petri dishes were maintained at 4°C for 30 min to fix the proteins in the disks and then incubated at 28°C or 30°C (depending on the bacterium type) for 16 hr. In order to determine the antimicrobial activity of fusion proteins, the diameter of inhibition zones surrounding the disks was measured in triplicates in millimeter (*r*_zone_ − *r*_disk_). It must be mentioned that the gentamicin disk (10 μg/disk) was used as the positive control and the proteins extracted from the non‐transgenic HRs as the negative control. Statistical analysis of the treatments was conducted using MSTATC and SAS 9.1 softwares in a factorial experiment using a completely randomized design with three replications. If there were significant differences between the treatments, Duncan's multiple range test at the relevant significant level was used to compare the means.

### 2.8 Minimum inhibitory concentrations of the recombinant proteins

The microdilution method was used to determine the minimum inhibitory concentrations (MICs) of the recombinant proteins according to Che et al. (2011). Briefly, five different protein concentrations (5.62, 11.25, 22.5, 45, and 90 μg/ml) of the purified recombinant proteins were tested against bacterial suspensions in 96‐well plates with serial dilutions in a final volume of 200 μl. Twenty microliters of the bacterial suspensions (1.5 × 10^8^ cells) was added to each well and the plates were maintained at 28°C or 37°C for 24 hr. The MIC was measured by the lowest concentration with no visible growth. LB broth was used as the negative control and bacterial culture without addition of antimicrobial recombinant proteins was used as the positive control. All experiments were designed and performed in triplicates for each bacterial species (Che et al., 2011).

### 2.9 Antifungal activity of the recombinant proteins

Antifungal activity of the recombinant proteins was evaluated by mixing the recombinant proteins to the growth medium. Recombinant proteins were added in PDA medium to the final concentration of 50 μg/ml. Ten‐millimeter plugs of young fungal cultures were kept in the center of 9 cm PDA containing Petri plates. Fungal mycelia diameters were measured at 4–7 days after inoculation at 25°C. The following equation was used to calculate the percentage of mycelium growth inhibition:

C=W-T/W×100

where C is the percentage of mycelium growth inhibition, *W* is the radial growth zone diameter in the control, and *T* represents the diameter of the radial growth zone in the treatment.

For fungal MIC assessment, various concentrations (5, 10, 20, 30, 40, and 50 µg/ml) of the purified recombinant proteins were mixed with the fungal spore suspension (1 × 10^8^ cfu/ml) in a final volume of 100 µl. After 24 hr, germinated conidia and spores were counted under a light microscope. The MIC values were defined as the lowest concentration of recombinant proteins required for complete suppression of fungal spore and conidia germination (Yevtushenko et al., 2005).

### 2.10 Scanning electron microscopy analysis

*Alternaria alternata* (PTCC 5,224) and *Pythium *sp. fungi were cultured on the PDA medium for 4 days. Fungal plugs (1 cm^2^) were injected with each of the recombinant proteins (50 μg/ml) and incubated at 25°C for 24 hr followed by freezing at −80°C for 24 hr. The frozen plugs were then put in a freeze‐drying machine for 4 hr. The fixed samples were coated with gold nanoparticles using a Desk Sputter Coater–DSRI, and finally scanned with an electron microscope (FE‐SEM, Tescan Mira3 LMU, at HV = 20 kV).

## 3 RESULTS

### 3.1 Molecular analysis of transgenic plants

Transgenic HRs were produced and designated as CBD‐DrsB1‐XX and DrsB1‐CBD‐XX based on the type of the construct employed to express the recombinant proteins, XX represents the transgenic line number.

**Figure A1:**
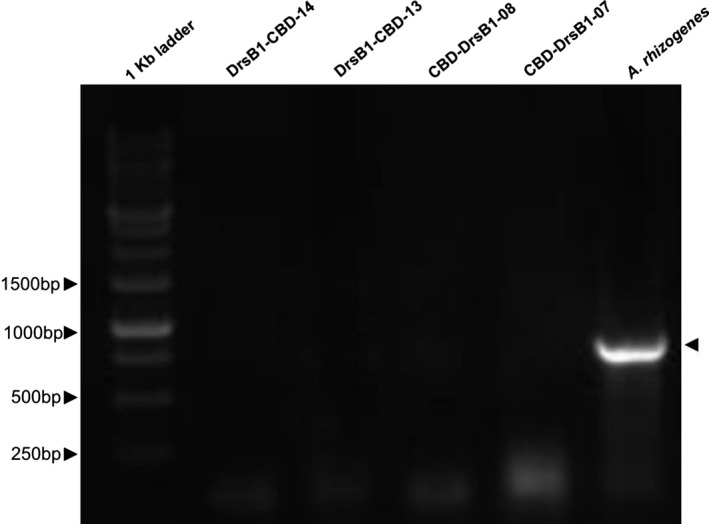
PCR analysis of tobacco hairy roots obtained after transformation with A. rhizogenes. DNA isolated from A. rhizogenes as positive control. Arrow shows amplified fragments of virG

Polymerase chain reaction analysis of putative transgenic HRs using *rol*C and DrsB1 peptide specific primers resulted in amplification of 600 bp and 100 bp PCR products, respectively, suggesting that HRs are transgenic, whereas no PCR product was amplified in non‐transgenic HRs. Moreover, PCR analysis using the *Vir*G specific primers did not lead to any amplification, ruling out possible *A. rhizogenes* contamination (Appendix Figure A1).

**Figure 2:**
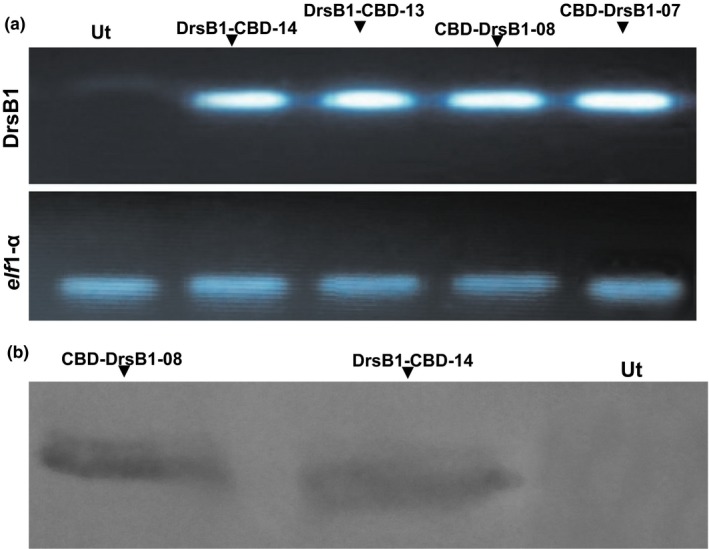
Semi‐quantitative RT‐PCR (a) and western blotting (b) analysis of the transgenic and control hairy roots (HRs). The polymerase chain reaction (PCR) products of the *elf*1‐α housekeeping gene were used to compare the level of mRNA transcripts of different transgenic HRs

Expression of the recombinant genes was determined using semi‐quantitative RT‐PCR analysis according to Nazarian Firouzabadi et al. (2007). A PCR product with the approximate 100 bp size was amplified from the transgenic HRs lines, suggesting that the recombinant genes are transcribed. No fragment was observed in the non‐transgenic HRs controls. Considering the intensity of mRNA transcript of both recombinant genes and the *elf*‐1α as the housekeeping gene, no obvious difference was noticed between different transgenic HR lines regarding the level of the expression of recombinant genes (Figure 2a). In the selected transformants, the presence of CBD‐DrsB1 and DrsB1‐CBD recombinant proteins was analyzed using Western blotting analysis. The recombinant proteins were produced in the HRs, whereas no traces of such proteins were found in the non‐transgenic HRs (Figure 2b).

### 3.2 Antimicrobial activity of the recombinant proteins

Sixty micrograms per milliliter (60 μg/ml) total protein from the CBD‐DrsB1‐8, CBD‐DB1‐14, and DrsB1‐04 lines (Alibakhshi, 2017) were used to compare the activity of recombinant proteins against four pathogenic bacteria. Total protein from transgenic HRs lines had a significant (*p* < 0.01) higher antibacterial activity than total protein extracted from non‐transgenic HRs. Interestingly, the total protein from transgenic HRs lines exhibited a higher activity than DrsB1‐04 transgenic line (Table 1).

**Table 1:** The effect of recombinant fusion proteins on four bacteria species. The inhibition zone diameter is presented as the mean ± standard deviation of three replicates

Diameter of the inhibition zone (mm)				
Transgenic line/antibiotics	*Agrobacterium tumefaciens*	*Pectobacterium carotovorum*	*Ralstonia solanacearum*	*Xanthomonas campestris*
CBD‐DrsB1‐08	13.33 ± 0.5^d^	21. 60 ± 0.6^ab^	7.60 ± 0.5^ef^	8.30 ± 0.5^e^
DrsB1‐CBD‐14	12.00 ± 0.5^d^	13.40 ± 0.6^d^	3.50 ± 0.3^gh^	2.10 ± 0.3^hi^
DrsB1‐04	3.60 ± 0.6^gh^	5.00 ± 0.5^fg^	1.20 ± 0.2^hi^	0^i^
Ut	0	0	0	0
Gentamicin (10 μg)	21.30 ± 0.5^ab^	22.30 ± 0.5^a^	18.80 ± 0.5^bc^	16.60 ± 0.5^c^

Means that do not share the same alphabetic superscript are significantly (*p* < 0.01) different according to Duncan's multiple range test.

Ut: non‐transgenic control line.

**Figure 3:**
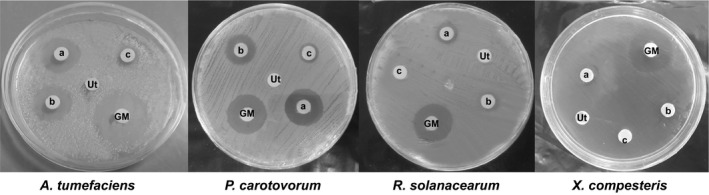
The antimicrobial activities of the total proteins derived from the transgenic and control hairy root lines. (a) CBD‐DrsB1‐08 line, (b) DrsB1‐CBD‐14 line, (c) DrsB1‐04 line, Ut: non‐transgenic control plant and GM: Gentamicin (10 μg/disk)

The antibacterial activity of the recombinant proteins was assayed for bactericidal activity against four gram‐negative bacteria. The recombinant proteins showed a stronger activity against *P. carotovorum* and *A. tumefaciens* bacteria than *R. solanacearum* and *X. campestris* bacteria (Figure 3). The recombinant protein of CBD‐DrsB1‐8 transgenic HR line demonstrated a stronger inhibition activity against *P. carotovorum *in comparison to that of DrsB1‐CBD‐14 transgenic line. Moreover, the inhibitory activity of the CBD‐DrsB1‐8 and DrsB1‐CBD‐14 lines was significantly (*p* < 0.01) higher than the DrsB1‐04 transgenic line (Table 1).

**Figure 4:**
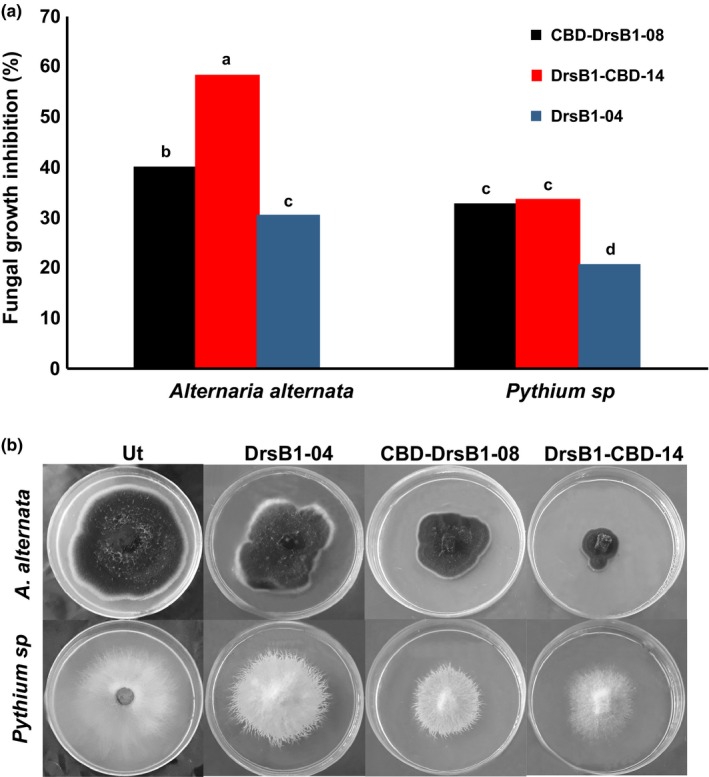
(a) The diagram showing the effect of recombinant proteins on fungal growth for *Alternaria alternata* and *Pythium *sp. (b) Antifungal activity of non‐transgenic control and recombinant proteins isolated from different transgenic HR lines. Ut: non‐transgenic control line

The antifungal activity of the recombinant proteins was assayed in triplicates against *A. alternata* and *Pythium *sp. fungi. Total protein isolated from transgenic HRs exhibited a significant (*p* < 0.01) antifungal activity against both fungi in comparison to non‐transgenic control HRs. Additionally, the inhibitory effect of the two recombinant proteins with the CBD was significantly (*p* < 0.01) stronger than that of the DrsB1‐04 peptide. Among the two recombinant proteins, DrsB1‐CBD exhibited a higher inhibitory effect against *A. alternata* than the CBD‐DrsB1 recombinant protein, whereas the two recombinant proteins had a similar inhibitory effect (*p* > 0.05) on *Pythium *sp. growth (Figure 4).

**Figure 5:**
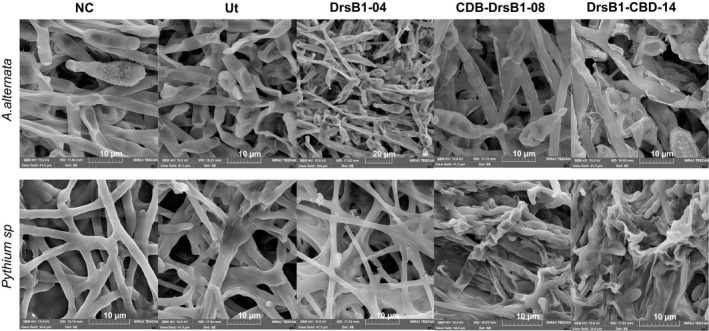
Electron microscopy images of *Alternaria alternata* (upper panel) and *Pythium *sp. (lower panel) hyphae treated with the recombinant proteins (50 μg/ml). Scale bars are indicated in µm for each image. Ut: non‐transgenic control line and NC: nontreated control

*Alternaria alternata* and *Pythium *sp. fungi were treated with the recombinant proteins to study effects of antifungal activity on the cellular structures of fungi. It was found that the growth and development of *A. alternata* hyphae slowed or stopped under the influence of the two types of recombinant proteins. SEM images demonstrated that *A. alternata* spores were deformed and shrunken, and apparently, their contents leaked out. As observed, the DrsB1‐CBD recombinant protein had greater destructive effects on this fungus spores than the CBD‐DrsB1 recombinant protein. Additionally, deformation and adhesion of *Pythium* sp. mycelia were observed. Both recombinant proteins had similar effects on the structure of *Pythium *sp. mycelia (Figure 5).

The MIC, the lowest concentration of recombinant fusion proteins inhibiting the pathogen growth, was determined in a microdilution assay. The MIC of the recombinant fusion proteins was in the range of 45–90 μg/ml, whereas a higher concentration of DrsB1 peptide (90 μg/ml) was needed to inhibit bacterial growth in vitro (Alibakhshi, 2017). The MIC value of recombinant fusion proteins for *P. carotovorum* and *A. tumefaciens *was lower (45 μg/ml) in comparison to the MIC value for *R. solanacearum* and *X. campestris* (90 μg/ml) bacteria. Interestingly, the MIC of the recombinant fusion proteins was significantly different for *A. alternata*, suggesting the position of DrsB1 peptide may have an effect in the overall activity of the recombinant proteins. A lower (10 µg/ml) concentration of the DrsB1‐CBD recombinant fusion protein was needed to completely inhibit the *A. alternata* conidia germination in comparison with that of CBD‐DrsB1 (20 µg/ml) recombinant fusion protein. Furthermore, a MIC of 30 µg/ml of the DrsB1 peptide was sufficient to inhibit *A. alternata *conidia germinations, suggesting that fusion of DrsB1 to the CBD enhanced the antifungal activity of DrsB1 peptide. Interestingly, DrsB1‐CBD (20 µg/ml) recombinant protein had a better antifungal activity against *Pythium *sp. than CBD‐DrsB1 (30 µg/ml) and DrsB1 (40 µg/ml).

## 4 DISCUSSION

Over the past few decades, various approaches have been employed in molecular biology to improve and increase plant resistance to a broad spectrum of plant pathogens (Cao, Li, & Dong, 1998). For instance, expression of fungal and bacterial cell wall‐degrading enzymes (chitinases), expression of pathogenesis‐related proteins, increase in production of host proteins and metabolites involved in plant defense pathways, and expression of plant antimicrobial proteins and peptides have been reported (Punja, 2001).

All living organisms produce different classes of antimicrobial peptides as a part of their innate immune system to combat pathogens (Hancock & Scott, 2000; Holaskova, Galuszka, Frebort, & Oz, 2015). To enhance the antibacterial activity of natural peptides, researchers design and synthesize new variants or recombinant peptides for pharmaceutical and agricultural industries (Melo, Ferre, & Castanho, 2009; Yevtushenko & Misra, 2012; Yevtushenko et al., 2005). In this study, the DrsB1 peptide was fused to the CBD of the *Avr*4 gene from *C. fulvum* so that the recombinant protein could bind to the fungal cell wall as well as the peptidoglycans of bacterial cell walls perturbing the integrity of cell wall components. Although the DrsB1 peptide exhibited strong in vitro antimicrobial activity against bacteria, fungi, protozoans, and yeasts, the antibacterial activity was significantly increased when the N‐terminal region of the DrsB1 peptide and MsrA2 analog was manipulated to bind to the negatively charged lipids (Osusky et al., 2005). The expression of modified peptides in potato and tobacco plants led to the production of transgenic lines with enhanced resistance to a number of devastating plant pathogens. Interestingly, the recombinant proteins extracted from transgenic HRs in this study, exhibited a significant (*p* < 0.01) antibacterial activity against plant pathogenic gram‐negative bacteria. Similarly, Badrhadad, Nazarian‐Firouzabadi, and Ismaili (2018) provided strong evidence that fusion of an alfalfa antibacterial peptide to rice chitinase CBD inhibited the growth and development of plant pathogens (Badrhadad et al., 2018).

The inhibitory concentrations of the recombination proteins varied significantly for different bacteria and fungi. It was found that the fungi are more sensitive to recombinant proteins than bacteria. The susceptibility of bacteria and fungi to recombinant proteins may be attributed to the variation of different cell components present in bacteria and fungi (Marcos, Muñoz, Pérez‐Payá, Misra, & López‐García, 2008).

Although the mechanism by which antimicrobial peptides attack pathogens is not fully understood, the mode of action may involve interaction of charged components (Nguyen et al., 2011; Toke, 2005). A relatively higher positive charge of the recombinant peptides and the binding affinity of the CBD toward cell wall building blocks may accumulate more DrsB1 peptide on the surface of the pathogen leading to effective interaction between positively charged recombinant peptides and the negatively charged membrane surface of the pathogens. It is documented that the positive charge in the hydrophobic part of cationic peptides is essential for their antimicrobial activity (Yin, Edwards, Li, Yip, & Deber, 2012). In other words, fusion of the CBD from *Avr*4 along with addition of histidine residues increases the antimicrobial activity of the recombinant proteins against pathogenic microbes. There seems to be a relationship between the chitin‐binding capability and antimicrobial activity of the CY‐AMP peptide against the gram‐positive bacteria *Lactococcus lactis*, *Streptococcus mutans*, and *Clavibacter michiganensis*, the gram‐negative *Erwinia carotovora* and *Enterobacter cloacae*, and the fungi *Fusarium oxysporum* and *Geotrichum candidum* (Yokoyama et al., 2009). Mutations in the CBD of the CY‐AMP peptide led to a decrease in chitin‐binding ability and hence antifungal activity, whereas the antibacterial activity of CY‐AMP against gram‐positive and gram‐negative bacteria did not change in comparison with that of the wild‐type peptide (Yokoyama et al., 2009). It is noteworthy that the antifungal activity of a barley chitinase mutated at the important catalytic domain of acidic residues declined by 75% in comparison with that of wild‐type chitinase (Andersen, Jensen, Robertus, Robert, & Skriver, 1997). Overall, it can be concluded that the antifungal activity of the recombinant proteins increases as CBD shows intrinsic affinity for chitin. Therefore, the recombinant proteins in this study had a relatively higher inhibitory effect against *A. alternata* in comparison to *Pythium *sp. Furthermore, the presence of chitin in the cell wall seems to be crucial for antifungal activity of the recombinant proteins (Yan et al., 2008). The results of a similar study indicated that a protein bound to a chitin‐binding protein with antifungal activity from *Moringa oleifera* seeds (MO‐CBP3) had a strong antifungal activity against *Fusarium solani*, *F. oxysporum, *but did not inhibit the growth and germination of *Pythium oligandrum*, suggesting that adhesion to the chitin is vital for antibacterial activity (Gifoni et al., 2012).

In conclusion, results of the present study indicated that the cell wall chitin can be targeted to control plant pathogenic fungi containing chitin (Yan et al., 2008). Accumulation of DrsB1 peptide on the surface of fungal pathogens in a carpet‐like manner (Pouny, Rapaport, Mor, Nicolas, & Shai, 1992; Shai, 1999) may result in instability of the fungal cell, eventually leading to the fungal cell death (Figure 5). The DrsB1‐CBD recombinant protein had a higher inhibitory effect than CBD‐DrsB1 recombinant protein, suggesting that DrsB1 peptide may interfere with CBD affinity for cell wall chitin, leading to a lower concentration of the CBD‐DrsB1 recombinant protein at the cell wall surface. It is noteworthy that no plant chitinase has so far been identified with the CBD at the C‐terminal part of the main catalytic domains (Beintema, 1994; Iseli, Boller, & Neuhaus, 1993). Due to the high antimicrobial activity of the recombinant proteins of this study, it would be interesting to introduce the recombinant genes to crop plants and generate resistant lines to devastating plant pathogens.

## CONFLICT OF INTERESTS

None declared.

## AUTHORS CONTRIBUTION

MK performed the experiments. F.N‐F designed the experiments, wrote the manuscript. A.A and RSK helped in designing some of the experiments.

## ETHICS STATEMENT

None required.

## Data Availability

All data used in this study are presented in the manuscript.
